# Use of costic acid, a natural extract from *Dittrichia viscosa*, for the control of *Varroa destructor*, a parasite of the European honey bee

**DOI:** 10.3762/bjoc.13.96

**Published:** 2017-05-18

**Authors:** Kalliopi Sofou, Demosthenis Isaakidis, Apostolos Spyros, Anita Büttner, Athanassios Giannis, Haralambos E Katerinopoulos

**Affiliations:** 1 Department of Chemistry, University of Crete, Voutes, Heraklion, 71003, Crete, Greece; 2 Institut für Organische Chemie, Universität Leipzig, Johannisallee 29, 04103 Leipzig, Germany; 3 recent address: Department of Chemistry and Food Chemistry, TU Dresden, 01062, Dresden, Germany

**Keywords:** *Apis mellifera*, *Dittrichia viscosa*, natural products, *Varroa destructor*, varroosis

## Abstract

Costic acid has been isolated from the plant *Dittrichia viscosa* and its efficacy against *Varroa destructor*, a parasite of *Apis mellifera*, the European honey bee, has been studied. Costic acid exhibited potent in vivo acaricidal activity against the parasite. Initial experiments showed that the compound is not toxic for human umbilical vein endothelial cells (HUVEC) at concentrations of up to 230 micromolar (μM), indicating that costic acid could be used as a safe, low-cost and efficient agent for controlling varroosis in honey bee colonies.

## Introduction

The honey bee ectoparasite *Varroa destructor* (Anderson & Trueman) was discovered by Oudemans in 1904 [1]. The mite was located on the island of Java and was infesting *Apis cerana,* the Asiatic bee, which does not exhibit any symptoms when infested by this parasite. Oudemans named the mite *Varroa jacobsoni.* After extensive studies on mtDNA Co-I gene sequences and comparison of the morphological characters on numerous populations of *V. jacobsoni* world-wide, Anderson and Trueman came to the conclusion that the mite belongs to two species: a) *Varroa jacobsoni* s.s., located in the Malaysia–Indonesia region, infesting *Apis cerana* F. and b) *Varroa destructor,* Anderson & Trueman, infesting both, *Apis cerana* in Asia and *Apis mellifera* worldwide [2]. Given that *A. mellifera* is not tolerant to the mite, varroa can attack and eliminate whole bee colonies within a period of a few years [3–4]. The decimation of the bee population entails a negative impact on the global economy, given that honey bees are considered the economically most important pollinators of crop monocultures worldwide [5]. A recent article on the protection of pollinators stresses the need for “statistically robust monitoring programs for native bees”, especially when taking into account that some species have been designated as endangered [6]. In times when the global pollinator decline is apparent [7–8], control of varroosis has a significant impact on the maintenance of wild plant diversity, ecosystem stability, and crop production.

In Europe, many beekeepers used acaricides such as coumaphos and synthetic pyrethroids to keep mite populations under control [9–10]. Literature data indicated that a number of pesticide residues have been detected in honey samples [11–12]. Given that the parasite has developed significant resistance to synthetic acaricides, alternatives such as oxalic acid [13–15] were proposed and extensively studied for their effects against varroosis. The use of oxalic acid as acaricide against *V. destructor* was first proposed in 1989 [16]. Subsequent studies indicated the potential of the method [17–22], which has been applied with better results during the broodless period. Application of the method does not increase the amount of oxalic acid in honey [23–25] and has no toxic effects on bees. However, later studies indicated that the utilization of oxalic acid by either trickling or spraying has a detrimental effect on brood development when open brood is present, and is therefore not as safe as it has been assessed in the past [26–27]. Recent findings support the hypothesis that *V. destructor* associates with bacteria capable of degrading oxalic acid [28].

Crete is a Greek island located in the south-east of Europe. Given the great diversity in the plant species of the flora of Crete, the area is an excellent feeding place for bee colonies, and beekeeping has been a profitable practice of the local population since ancient years. *Dittrichia viscosa* (L.) W. Greuter (syn. *Inula viscosa* (L.) Aiton), an invading ruderal species of the Asteraceae family [29], is a herbaceous perennial plant which is widespread in the Mediterranean region, known in folk medicine to possess anti-inflammatory [30], antipyretic, and gastric antiulcerous effects [31–32]. Due to lack of competing flowers in Autumn, the plant attracts many pollinating insects including bees, wasps and some butterflies to feed on, an indication that the plant volatiles do not act as repellents on bees. The fact that *D. viscosa*, called by the natives “akoniza”, has been used by the local beekeepers as a means of controlling *varroosis,* prompted us to analyze the components of the plant in order to identify the active agent and study its efficacy against the parasite [33–34]. The use of natural products against varroosis is an eco-friendly approach to this severe problem. Field tests on the use of methanolic extracts of *Lepidium latifolium* and *Zataria multiflora* indicated that these preparations exhibited acaricidal activity against the mites [35]. However, there are no preceding data on the action of costic acid against varroa. In this publication we report the isolation and structure identification of costic acid as a component of *D. viscosa,* as well as in vivo and field studies providing strong evidence of the acaricidal action of the acid against *V. destructor.*

## Results and Discussion

**Figure 1:**
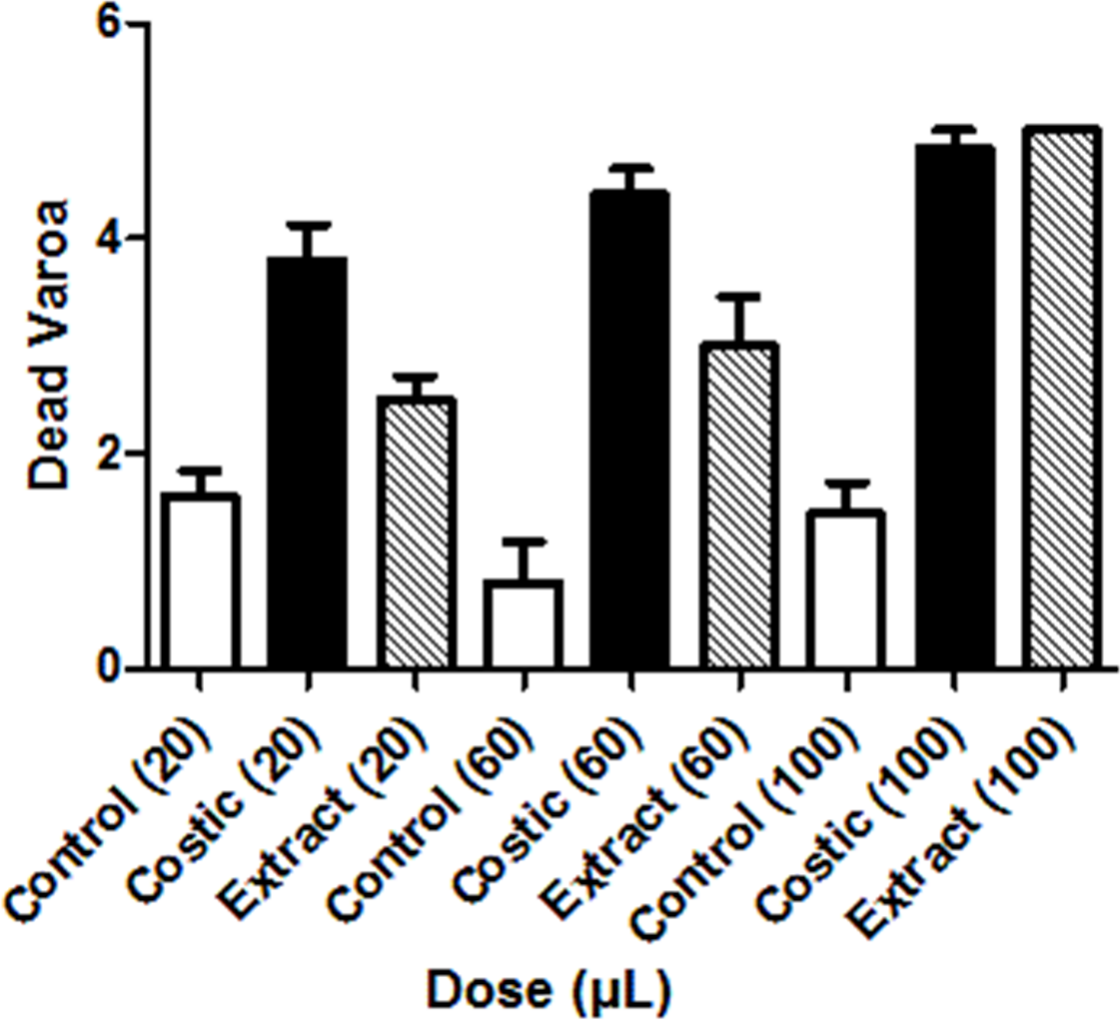
Mortality of *V. destructor* (number of mites) after 12 h treatment with 20, 60, or 100 μL dose of acaricide. The number in parenthesis indicates the microliters of solution used in each experiment. “Costic” indicates the use of costic acid solution and “extract” refers to the solution of the total methanol extract. Each vial, including controls, contained five mites. In control experiments, an equal number of microliters of acetone were added to the system. In all cases the solvent was allowed to evaporate before covering the vials.

**Screening tests**. Initial screening of the three *D. viscosa* fractions (A, B, and C, isolated by column chromatography, see Experimental) indicated that activity was apparent in the fraction with *R*_f_ = 0.80–0.60 (fraction B), whereas the rest of the fractions gave mite mortality rates identical to those of the controls. In the second series of screening tests, the two flavonoid-type components (7-O-methylaromadendrin and 7-O-methylaromadendrin-3-acetate) from fraction B were isolated, tested, and found inactive. However, the major component, namely costic acid, in its pure form, exhibited a higher efficacy against varroa than that observed in experiments using equal concentrations (mg/mL) of the total extract. Dose-dependent studies showed that after twelve hours, costic acid (42.7 mM), in the 60 microliter dose, is distinctly more active than the extract, whereas varroa mortality in the controls (see Materials and Methods) is approximately one third of that caused by costic acid (Figure 1).

**Figure 2:**
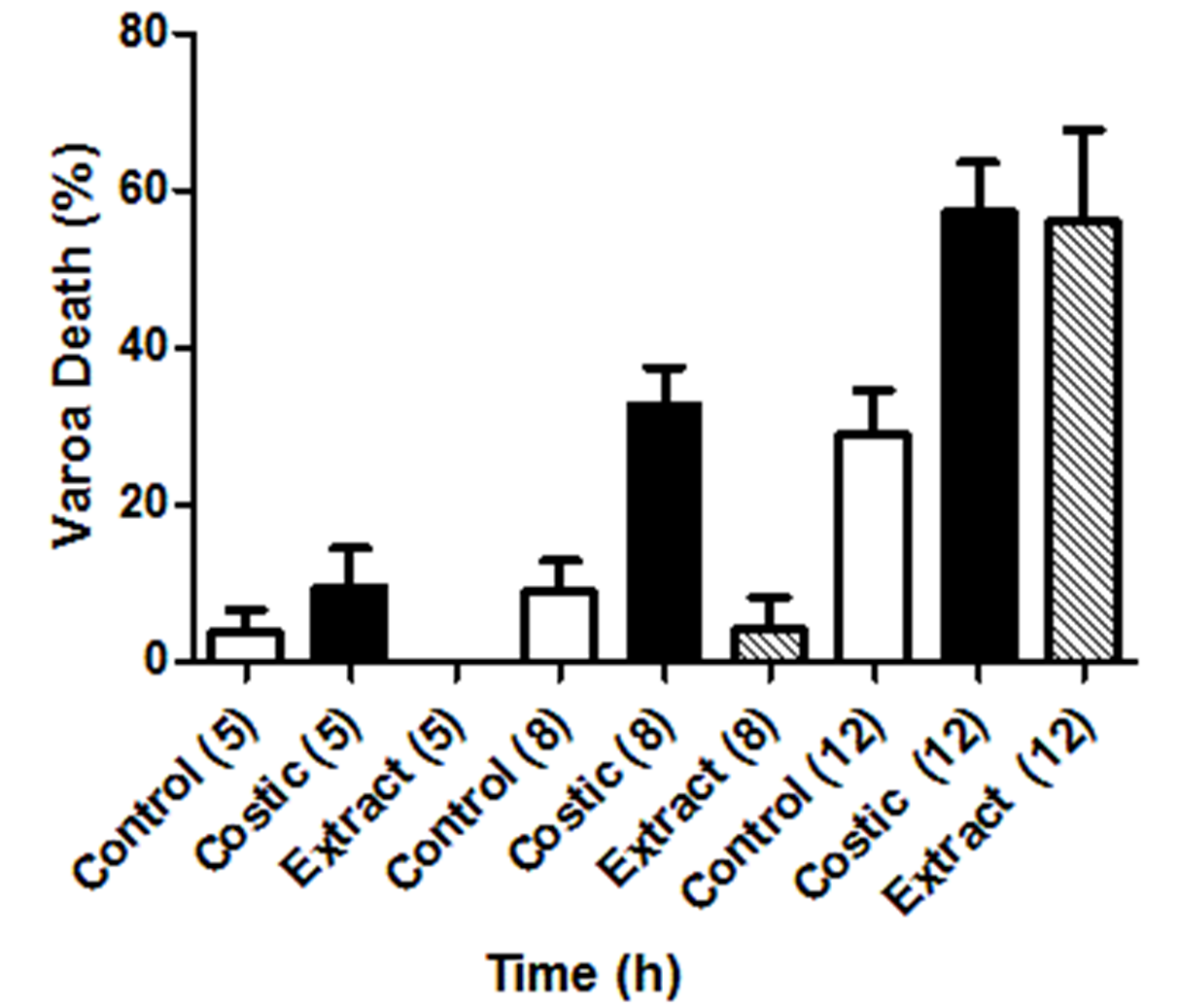
Time dependence of the mortality (%) of *V. destructor* after a 60 μL dose. The number in parenthesis indicates the number of hours that the mites were exposed to the acaricides. “Costic” indicates use of costic acid solution and “extract” refers to the solution of the total methanol extract. In control experiments, an equal number of microliters of acetone were added to the system. In all cases the solvent was allowed to evaporate before covering the vials.

Figure 2 shows the time dependence of the mortality (%) of varroa after a 60 microliter dose of costic acid or extract solution as compared to the controls. The varroa mortality is apparent after the first five hours. The activity of costic acid is more pronounced than that exhibited by the extract, which at eight ours remains at control level. After twelve hours, the varroa mortality reaches a value of 60%, similar to that of the extract. The varroa mortality in the control experiment was 30%. It must be stressed that there is a time-dependence of varroa mortality in the control experiments that reaches 100% after 19 hours, since varroa cannot survive for a longer time once being separated from its host.

**Field tests on bee populations**. Two field tests were conducted using 20 beehives on each trial at various locations on the island of Crete at the Lassithi Plateau and the Messara Plains. In the first field test (A), “group B” extract was used in a solution of 1 g of this extract in 1 mL ethanol that was further diluted with deionized water to a total volume of 1 L. In the second field test (B) a solution was prepared using 1 g of “group B” mixture diluted in sugar suryp 1:1 instead of deionized water. To allow for a comparison each bee population was divided into four groups. Two of these groups were treated with commercial acaricides. A fourth, the control group, was sprayed with deionized water. As commercial acaricide preparations were used a) bayvarol (active ingredient: flumethrine) and b) a 4% solution of oxalic acid in 50% sugar solution. The total volume of solutions that were sprayed directly on the bees per beehive was 200 mL for the extract and 70 mL in the case of the oxalic acid solution. Dead varroa mites were collected on a sheet of paper covered with vaseline and sunflower oil. After 48 h, the number of dead varroa mites was counted.

**Figure 3:**
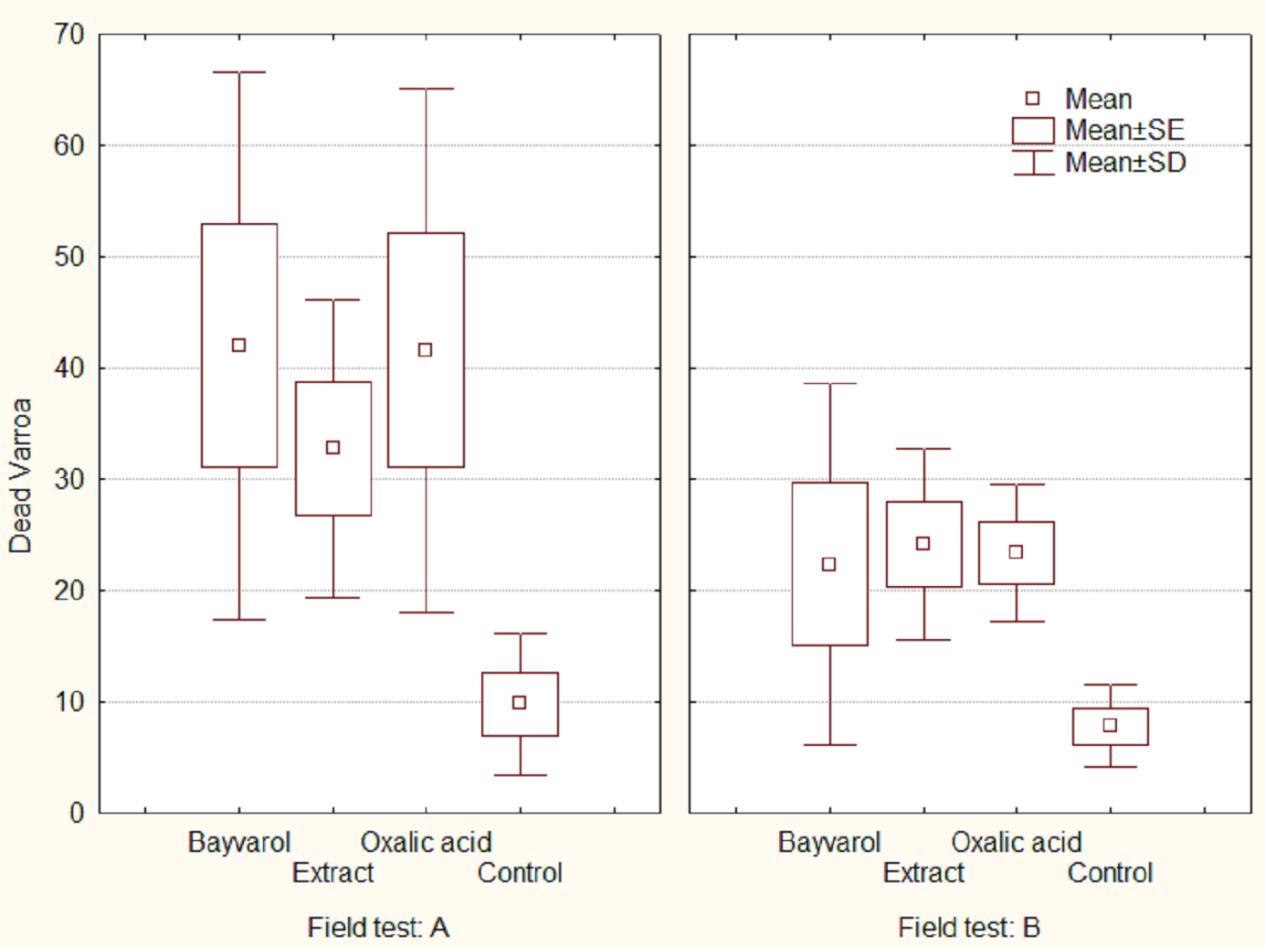
Field test results on application of extract B, bayvarol and oxalic acid to bee populations.

The results and the statistical analysis of the two field tests are presented in Figure 3. The extract treatment was significantly different from the control in both field tests (*p* < 0.01), and was found to be equally efficient in affecting varroa mortality compared to the two commercial acaricides in field test B, while in field test A the extract showed 80% effectiveness compared to commercial acaricides. None of the above mentioned trials caused the death of treated bees.

**Figure 4:**
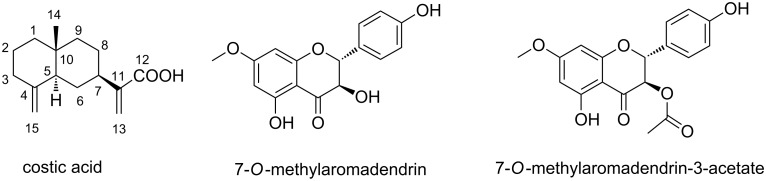
Structures of costic acid, 7-O-methylaromadendrin and 7-O-methylaromadendrin-3-acetate.

**Identification of the active component**. Spectroscopic data of the active component of *D. viscosa* indicated that it was a known compound, with the common name costic acid. A literature search indicated that there were discrepancies in the reported ^13^C NMR spectral data of costic acid [36–38] and given the importance of the compound in our biological studies, we decided to perform a full spectroscopic identification of the compound which is briefly described as follows: IR spectroscopy clearly indicated the presence of a carboxyl moiety. ^1^H NMR experiments indicated the presence of a methyl group and four olefinic protons, indicating the presence of two double bonds in the carbon framework. The mass spectrum gave a parent peak at *m*/*z*: 234 corresponding to a possible molecular formula of C_15_H_22_O_2_. With an unsaturation degree of five, one carbonyl and two olefinic double bonds present, we propose as a possible structure the bicyclic system of costic acid shown in Figure 4. We applied the same numbering as was used in previous publications to allow for an easier comparison.

^13^C NMR experiments showed the presence of four quaternary carbons at 36.04 (C-10), 145.44 (C-11), 150.73 (C-4), and 172.83 (C-12) ppm. The tertiary carbons appear at 50.00 (C-5) and 39.48 (C-7) ppm as shown by a DEPT 135° experiment, and the eight secondary carbons appear at 23.58 (C-2), 27.44 (C-8), 30.12 ppm (C-6), 36.96 (C-1), 41.19 ppm (C-9), 41.97 ppm (C-3), 105.63 (C-15) and at 124.94 (C-13) ppm. The fact that the last two secondary carbons belong to olefinic systems implies the presence of two exocyclic double bonds. Finally, the methyl group carbon appears at 16.51 ppm (C-14). The results of a detailed NMR spectroscopic analysis are shown in Table 1. The discrepancy in the literature data refers to carbons C-7 and C-9 which are assigned to resonances at 41.78 ppm and 39.31 ppm, respectively. In fact, as indicated in the DEPT 135° experiment, C-7 appears at 39.48 ppm and C-9 at 41.97 ppm and give signals of a tertiary and a secondary carbon, respectively. Concerning the absolute configuration of costic acid, we propose that it is the same as that reported by Bawdekar et al. [39] as indicated by a value of [α]_D_ = + 24.03 (*c* 1.3, MeOH) ([α]_D_ = + 23.42 (*c* 1.3, MeOH) [39]).

**Table 1:** NMR spectroscopic data of costic acid^a^.

Position	Proton	δ^1^H	COSY	NOESY	δ^13^C	HMBC

1	H-1 H-1΄	2.30 (m) 2.01 (q)	H-1’, Η-2 H-1, Η-3	H-9 H-9’, Η-3’	36.96	Η-5’, Η-9’, Η-3’
2	H-2 H-2΄	1.58 (m) 1.52 (m)	Η-2’ Η-3’, Η-2	Η-14	23.58	Η-1’, Η-3’
3	H-3 H-3΄	1.59 (m) 1.34 (m)	Η-1’ Η-2’	Η-5’, Η-7’	41.97	Η-1, H-14, H-2
4	–	–	–	–	150.73	H-1, H-1’, Η-5’, Η-3, Η-6’
5	H-5’	1.89 (dd)	Η-6’	Η-7’, Η-3’, Η-9’	50.00	Η-1’, Η-1, Η-2’, Η-9’, Η-14, Η-6’
6	H-6 H-6’	1.66 (m) 1.22 (m)	Η-6’ Η-6, Η-7’, Η-5’	Η-7‘, Η-5‘ Η-8‘, Η-14, H-9, H-15_α_	30.12	Η-8‘, Η-5‘, Η-9‘
7	H-7’	2.53 (m)	Η-6’, Η-8’	Η-6, Η-8 , Η-5’	39.48	Η-6’, Η-8’
8	H-8 H-8’	1.61 (m) 1.46 (m)	Η-9΄, Η-8’ Η-8 , Η-9’	Η-9’, Η-7’ Η-9, Η-6’, Η-14	27.44	Η-6’, Η-6
9	H-9 H-9’	1.59 (m) 1.28 (m)	Η-9’ Η-8’, Η-9	Η-1 Η-8, Η-6, Η-5’, Η-7’	41.19	Η-1’, Η-14
10	–	–	–	–	36.04	Η-5’, Η-3, Η-8, Η-14
11	–	–	–	–	145.44	Η-7’, Η-6, Η-6’, Η-8’
12	–	–	–	–	172.83	Η-7’
13	H-13_α_ H-13_β_	6.31 (s) 5.68 (s)	Η-13_β_ Η-7’, Η-13_α_	Η-7’,Η-6’,Η-8’, H-8	124.94	Η-7’, Η-6, Η-8
14	H-14	0.75 (s)	Η-13_α_, Η-13_β_	Η-2΄,Η-8΄,Η-6΄	16.51	Η-9, Η-9’, Η-1’, Η-1
15	H-15_α_ H-15_β_	4.39 (d) 4.71 (d)	Η-5’ Η-5’	Η-2’, Η-6’, Η-15_β_ H-15_α_	105.63	Η-5’, Η-1’, Η-1

^a^Protons at pseudoaxial positions are notified with (’).

Costic acid is a known natural product. It has been previously isolated from *D. viscosa* and its structure was confirmed via NMR analysis [36–38]. Moreover, *D. viscosa* is not the only species containing costic acid as a metabolite. It has been reported in the literature as a component in other plants such as *Nectandra cissiflora*, Nees [40], *Ferula communis* L [41], *Laggera pterodonta* (DC.) Benth [42], *Nectandra membranacea* (Sw.) Griseb [43], and *Stevia polyphylla* DC [44]. It has also been isolated from another species of *Dittrichia*, namely, *Dittrichia graveolens* (L.) Greuter [45]. The first study on the structure and the absolute configuration of the compound is from a report on the isolation of the acidic fraction of costus root oil [39]. The plant extracts exhibit a spectrum of biological activities [46]. *D. viscosa* extracts exhibited abortifacient, anti-implantational and luteolytic effects on rats [47]. The acidic components of its essential oil were found active against *Staphylococcus epidermidis*, *Streptococcus foecalis* and *Proteus vulgaris* [36]. The methanol extract of the aerial parts of the plant showed antioxidant activity in a study on lipid peroxidation of rat liver microsomes [30]. *n*-Hexane extracts from air dried *D. viscosa* young shoots exhibited strong fungitoxic effects [41]. Costic acid isolated from cypress pine (*Callitris glaucophylla*, Thompson et Johnson) exhibited termite-repellent activity [37]. In a study of its anti-inflammatory effects on the HaCaT keratinocyte cell line, costic acid significantly inhibited TNF-α and IFN-γ-stimulated chemokine production [48]. Costic acid exhibited selective cytotoxic effects to *Spodoptera littoralis*-derived Sf9 cells [49]. In spite of the diversity in biological activity, however, no acaricidal activity of either costic acid or *D. viscosa* extracts against *V. destructor* has been reported to date.

## Conclusion

Costic acid, isolated from the plant *Dittrichia viscosa* was tested for its efficacy against *Varroa destructor,* a parasite of the European honey bee. Costic acid as well as the total extract were active against the parasite. Field tests strongly suggest that this treatment does not have a toxic effect on the host. The fact that the compound is also not toxic against human umbilical vein endothelial cells (HUVEC) indicates that it could be used as a safe, environment friendly, and low-cost efficient agent of controlling varroosis in *Apis mellifera* colonies.

## Experimental

### Materials and methods

**Mites and bees**. The mites (*V. destructor*) were collected from colonies of *A. mellifera* with sister queens. For mite collection two different methods were used: In method A an apparatus introduced by Ariana et al. [50] was employed. Approximately 1000 infected adult honey bees were transferred directly from bee frames into a wire-screen cylinder. Then the cylinder was placed inside a second Plexiglas cylinder and CO_2_ was released for 5 min with a flow rate of 5 L/min to anesthetize the mites and the bees. The whole apparatus was then shaken several times to separate the mites from the bees. The inner cylinder was taken out of the apparatus to return the bees to their mother colony, a few minutes after they had recovered from the effect of the anesthesia. With this procedure more than 80% of mites were separated from the bees and fell to the bottom of the outer cylinder. The bottom lid of the outer cylinder was taken off and the mites were removed with soft brushes under a stereoscope and placed into the test vials. The second method involved removal of the mites from infected adult bees using a soft brush with the help of a stereoscope. In either case, no mortality of bees was observed after repeated trials and the mortality of mites observed was less than 0.1%.

**Plant material and extracts**. The leaves of *D. viscosa*, collected from five different areas of Crete, were dried in a shaded and dry place for twenty days. The plant was identified by botanist Dr. N. Roditakis, National Agricultural Research Foundation. Voucher specimens have been deposited at the Plant Protection Institute of National Agricultural Research Foundation (Heraklion Crete, Greece). A sample (10.3 g) was then pulverized and subsequently extracted with 1 liter of ethanol in a Soxhlet apparatus for 4.0 hours. The yield of the crude extract (2.70 g) was 26% based on the dry-leaves weight. The extract components were partially separated by gradient flash chromatography using silica gel 60 (0.040–0.063 mm) as absorbent and 5–80% of acetone in petroleum ether as eluent, followed by a washing of the column with methanol to remove the polar components. Three fractions were obtained with the following TLC (silica gel 60, F254S) *R*_f_-values: 0.90–0.80 (group A), 0.80–0.60 (group B) and 0.10–0.01 (group C). Each was tested in the in vivo assay. Biological activity was detected in the *R*_f_ 0.80–0.60 fraction. Three compounds were isolated from this fraction using the chromatographic methods described above. The major one (1.17% based on the dry-leaves weight) proved to be costic acid by NMR and MS techniques, while the other two were tentatively identified as the flavonoids 7-O-methylaromadendrin and 7-O-methylaromadendrin-3-acetate (Figure 4) by GC–MS analysis. To the best of our knowledge, these structures are reported for the first time as components of *D. viscosa.*

The conditions of the GC–MS experiment were as follows [51]: The essential oil and extracts were analyzed by GC–MS on a Shimadzu GC-17 A gas chromatograph coupled with a Shimadzu GC–MS-QP 5050 mass selective detector. A Supelco SBP-5 fused silica capillary column of 30 m, 0.25 mm i.d. (0.25 μm film thickness) was used for the analysis. The carrier gas was helium (He) at a flow rate of 0.9 mL/min. The injector and the detector were maintained at 250 and 230 °C, respectively. The column temperature was programmed as follows: 50 °C for 2 min, the temperature increased to 150 °C at a rate of 10 °C/min, increased to 290 °C at a rate of 4 °C/min, and then steadily held at 290 °C for 20 min. This oven program resulted in the best component resolution with a total analysis time of 67 min. The mass unit conditions were: ionization energy 70 eV, ion source 195 °C, with 0.5 scans/s from 35 to 450 *m/z*.

Chromatographs and mass spectra were recorded using the CLASS 5000 program. Components were identified on the basis of their mass spectra using the NIST 64 and NIST 120 GC–MS libraries and the comparison of their retention times with those of reference.

**Screening tests**. The experiment was conducted in a completely randomized design under laboratory conditions in five replications. In each replication 20, 60, or 100 microliter aliquots of a stock solution made of 10 mg of the total plant extract/1.0 mL acetone were placed on filter paper fitted to the caps of 20 mL vials, each containing five living mites. Mites were placed on the bottom of the vials to avoid direct contact with the filter paper. The same experiment was repeated using the same amounts of either group A, group B or group C (column chromatography fractions, see “Plant Material and Extracts”), or isolated pure costic acid. In the latter case the concentration of costic acid in the stock solution was calculated as 42.7 mM. Acetone was used in the control experiments which were performed in five replicates. In both cases the solvent was allowed to evaporate before covering the vials. The mortality of mites was recorded under a stereoscope binocular set after 5, 8, and 12 hours.

**Data analysis.** All graphs and statistical analysis (one way ANOVA followed by the Neuman-Keuls test) were performed with GraphPad Prism (v. 5.03) [52].

**Cytotoxicity assays**. The cytotoxicity of costic acid was investigated in human umbilical vein endothelial (HUVE) cells (PromoCell) by incubation with the cell proliferation reagent WST-1 (Roche, Mannheim, Germany) as previously described [53]. Briefly, 7500 cells per well (Greiner Bio-one, 96 well microplate, white, μclear bottom) were exposed to different compound concentrations (100 μL/well, containing less than 0.5% DMSO (v/v)) for 24 h before adding the WST-1 reagent. After 24 h of incubation the absorbance (440 nm and 650 nm) was measured according to the test protocol. Costic acid was not cytotoxic at concentrations of up to 230 micromolar (μM).

## Supporting Information

File 1 NMR, IR and MS spectra of costic acid.
