# Supplementary material for: Use of costic acid, a natural extract from Dittrichia viscosa, for the control of Varroa destructor, a parasite of the European honey bee
Source: Beilstein J Org Chem. 2017 May 18;13:952–9. doi: 10.3762/bjoc.13.96 (PMC5480341; doi:10.3762/bjoc.13.96)

Supporting Information

for

Use of costic acid, a natural extract from *Dittrichia viscosa*, for the control of *Varroa destructor*, a parasite of the European honey bee

Kalliopi Sofou[1], Demosthenis Isaakidis[1], Apostolos Spyros[1], Anita Büttner[2,3], Athanassios Giannis*[2] and Haralambos E. Katerinopoulos*[1]

Address: [1]Department of Chemistry, University of Crete, Voutes, Heraklion, 71003, Crete, Greece, [2]Institut für Organische Chemie, Universität Leipzig, Johannisallee 29, 04103 Leipzig, Germany and [3]recent address: Department of Chemistry and Food Chemistry, TU Dresden, 01062, Dresden, Germany

Email: Athanassios Giannis* - giannis@uni-leipzig.de; Haralambos E. Katerinopoulos* - kater@chemistry.uoc.gr

* Corresponding author

NMR, IR and MS spectra of costic acid

Table of contents:

^{1}H NMR of costic acid	S2
^{13}C NMR of costic acid	S3
DEPT 45°, 90° and 135° spectra of costic acid	S4
COSY spectrum of costic acid	S5
COSY spectrum of costic acid (0.0–4.5 ppm)	S6
HMBC spectrum of costic acid	S7
HMBC spectrum of costic acid (^{13}C: 0–65 ppm; ^{1}H: 0.0–2.6 ppm)	S8
HMQC spectrum of costic acid	S9
HMQC spectrum of costic acid (^{13}C: 0–65 ppm; ^{1}H: 0.0–2.6 ppm)	S10
NOESY spectrum of costic acid	S11
NOESY spectrum of costic acid (0.7–3.1 ppm)	S12
FTIR spectrum of costic acid	S13
Mass spectrum of costic acid	S14

^{1}H NMR of costic acid

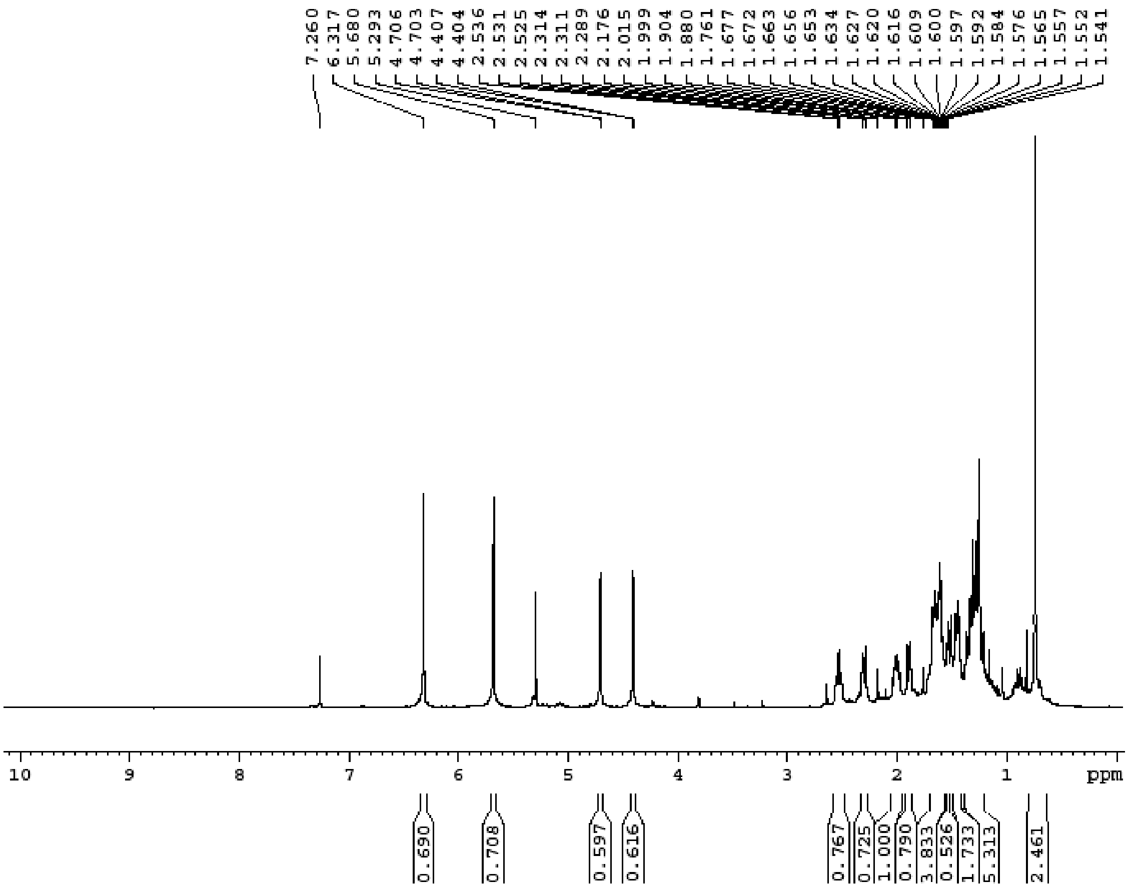

^{13}C NMR of costic acid

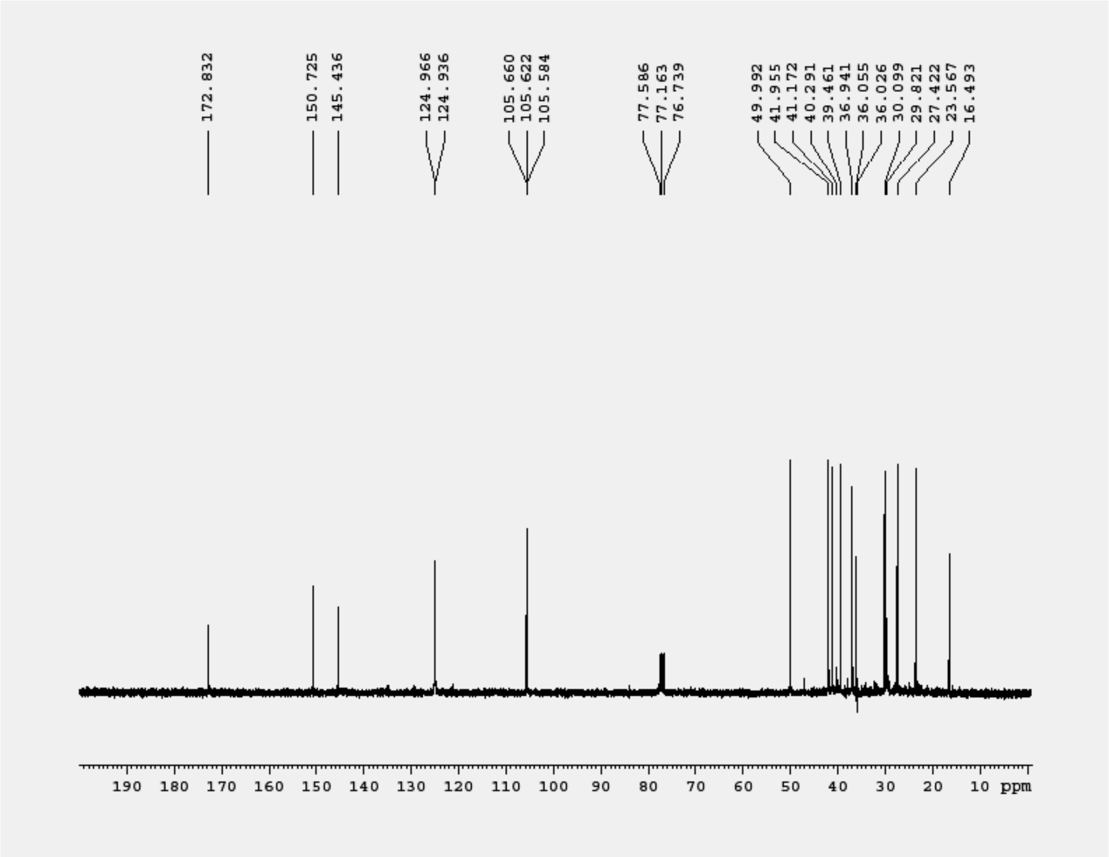

DEPT 45°, 90° and 135° spectra of costic acid

Dept 45^0

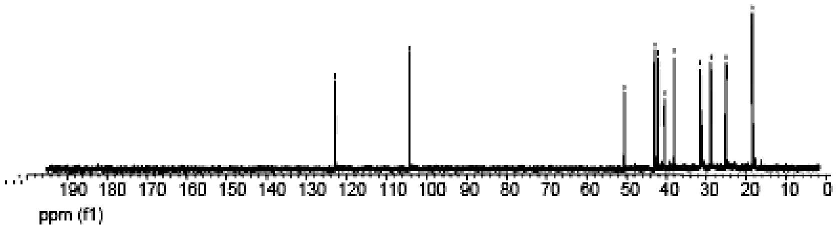

Dept 90^0

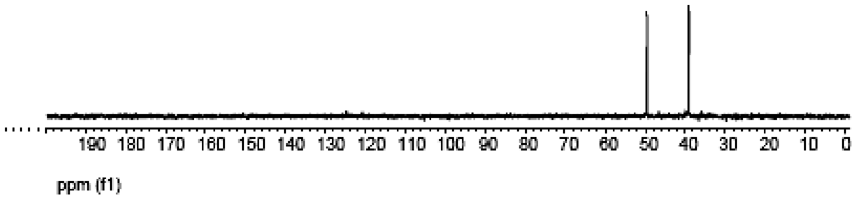

Dept 135^0

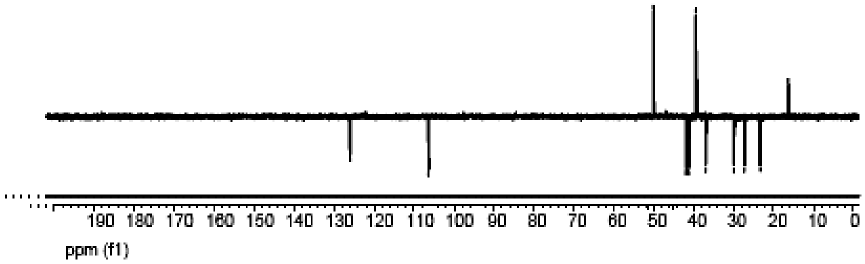

^{13}C

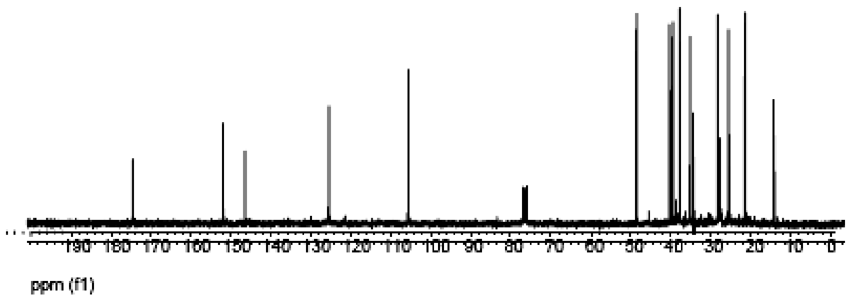

COSY spectrum of costic acid

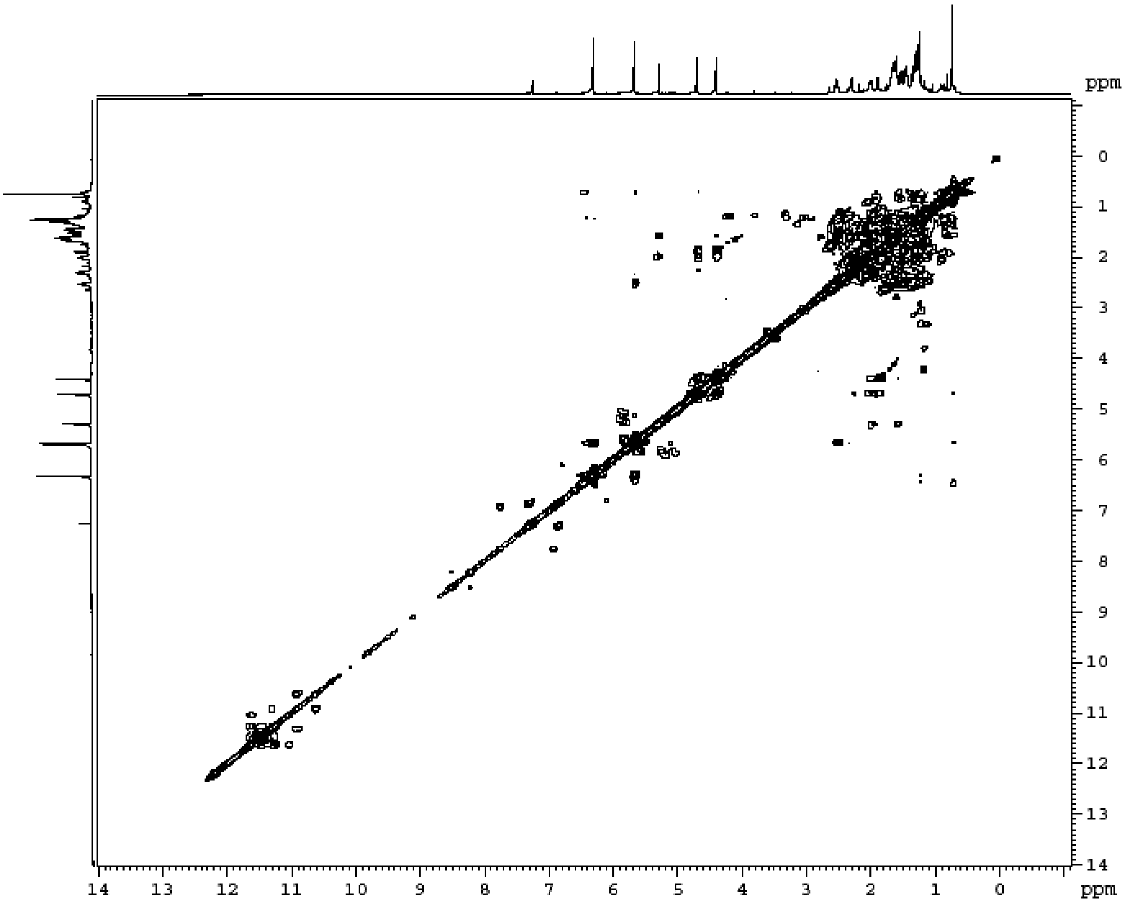

COSY spectrum of costic acid (0.0–4.5 ppm)

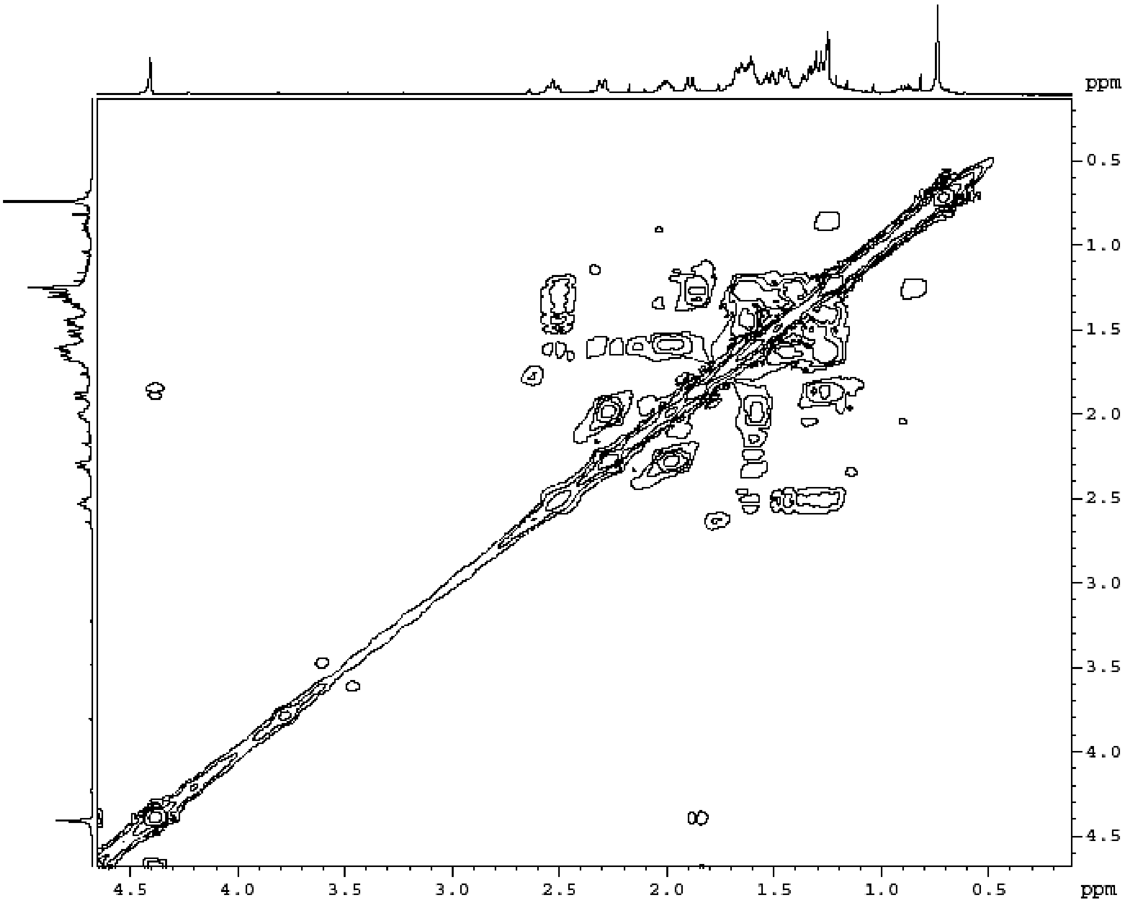

HMBC spectrum of costic acid

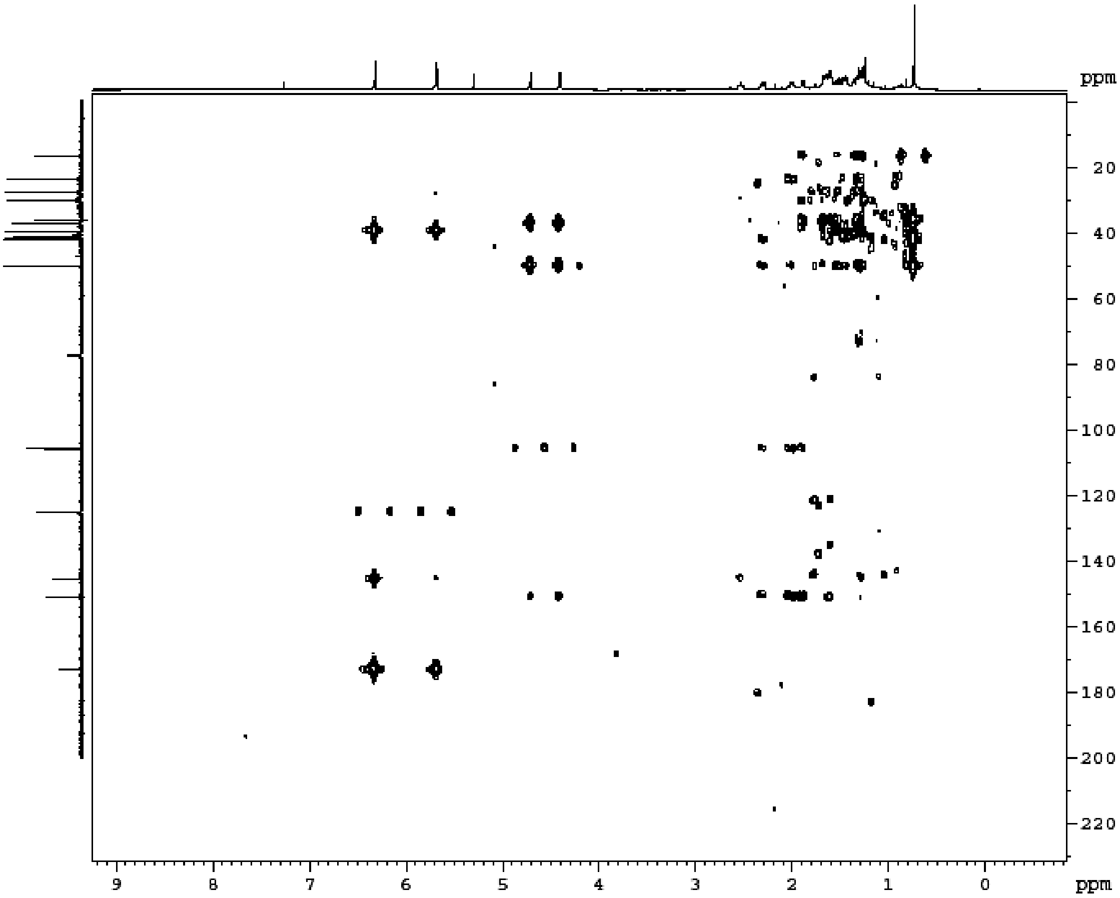

HMBC spectrum of costic acid (^{13}C: 0–65 ppm; ^{1}H: 0.0–2.6 ppm)

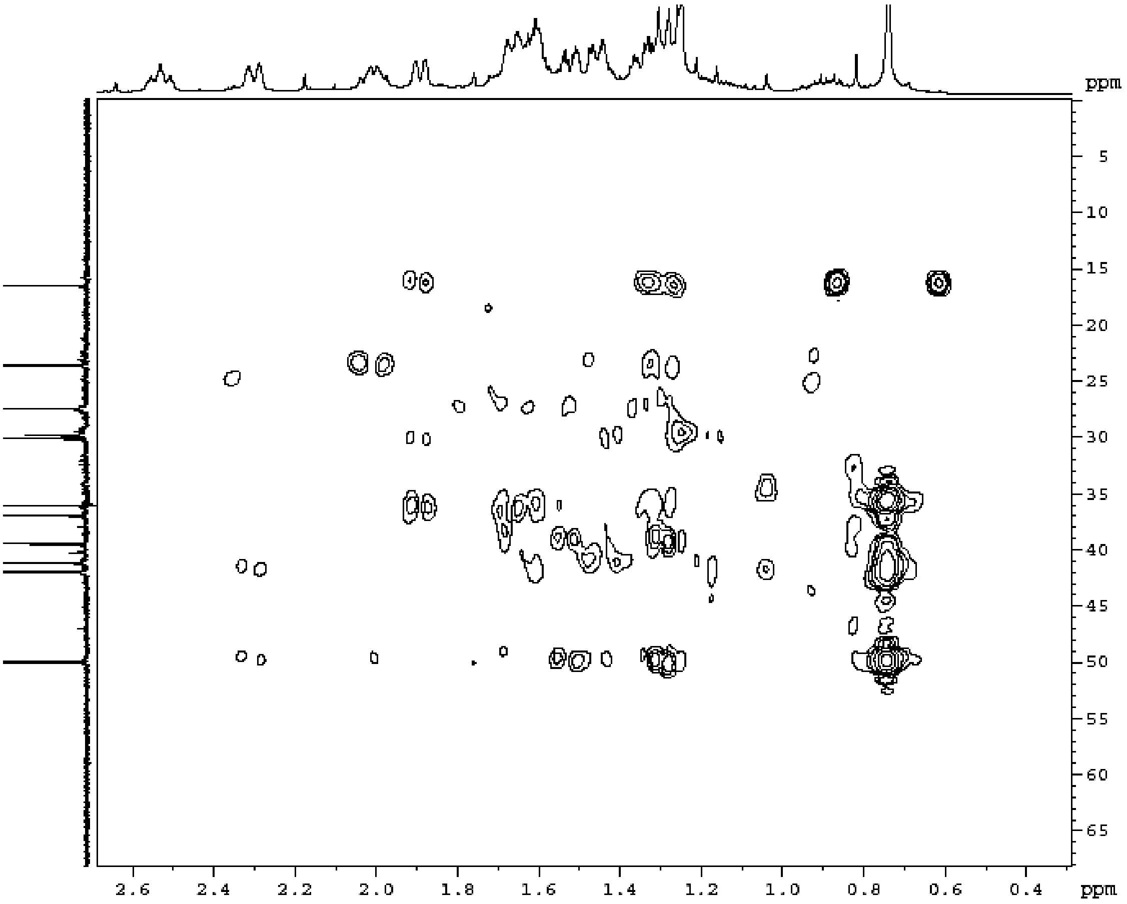

HSQC spectrum of costic acid

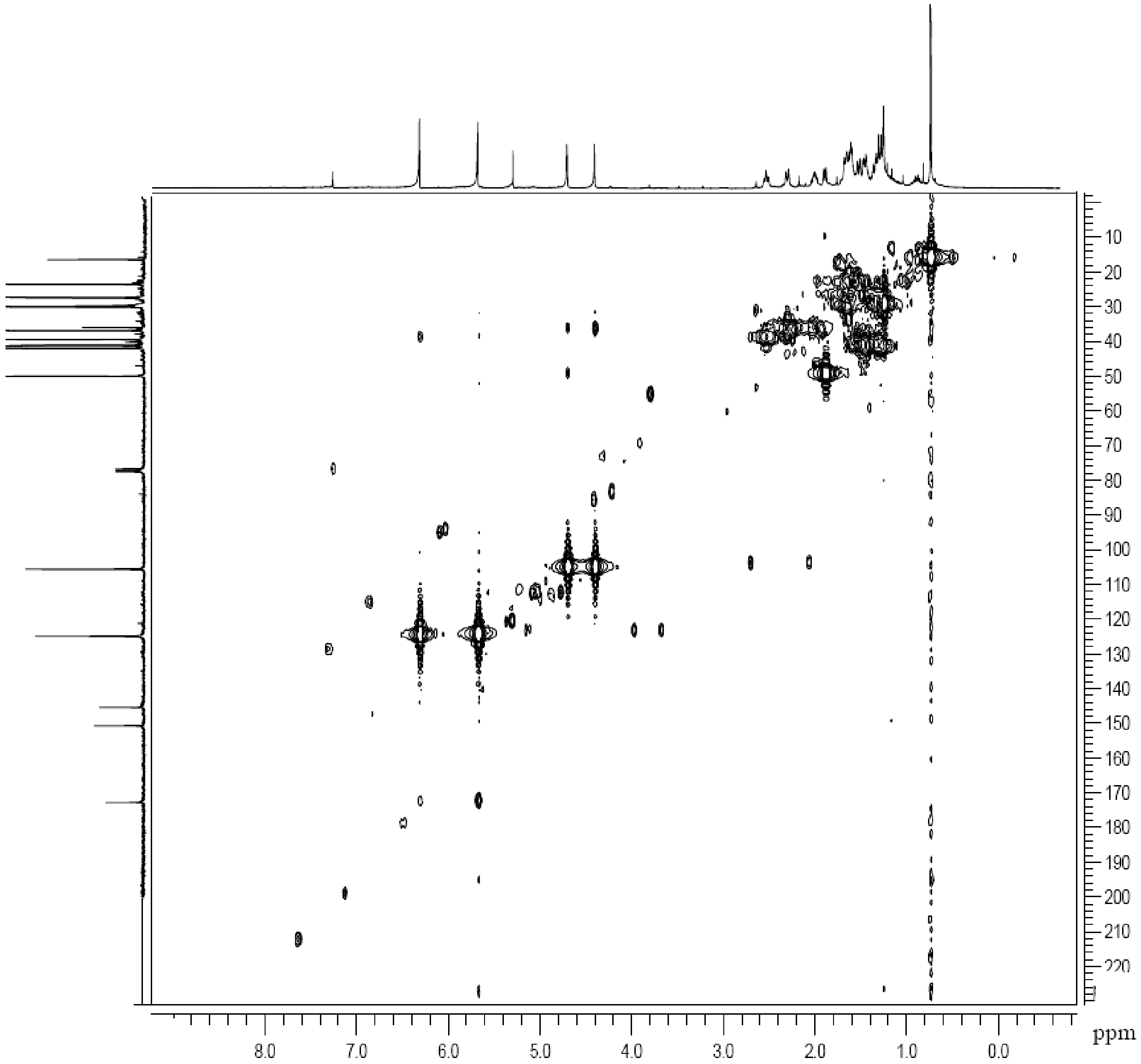

HSQC spectrum of costic acid (^{13}C: 0–65 ppm; ^{1}H: 0.0–2.6 ppm)

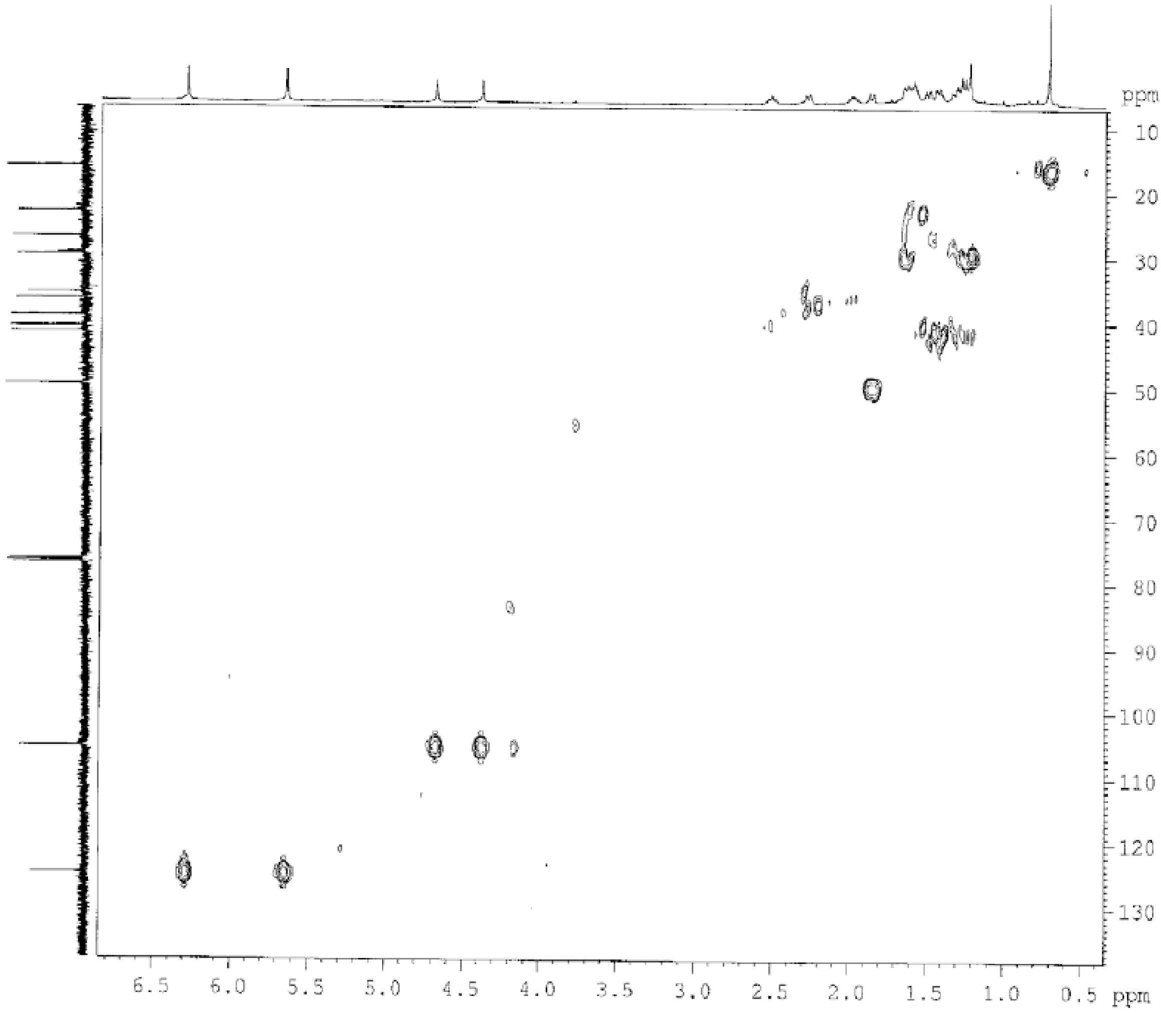

NOESY spectrum of costic acid

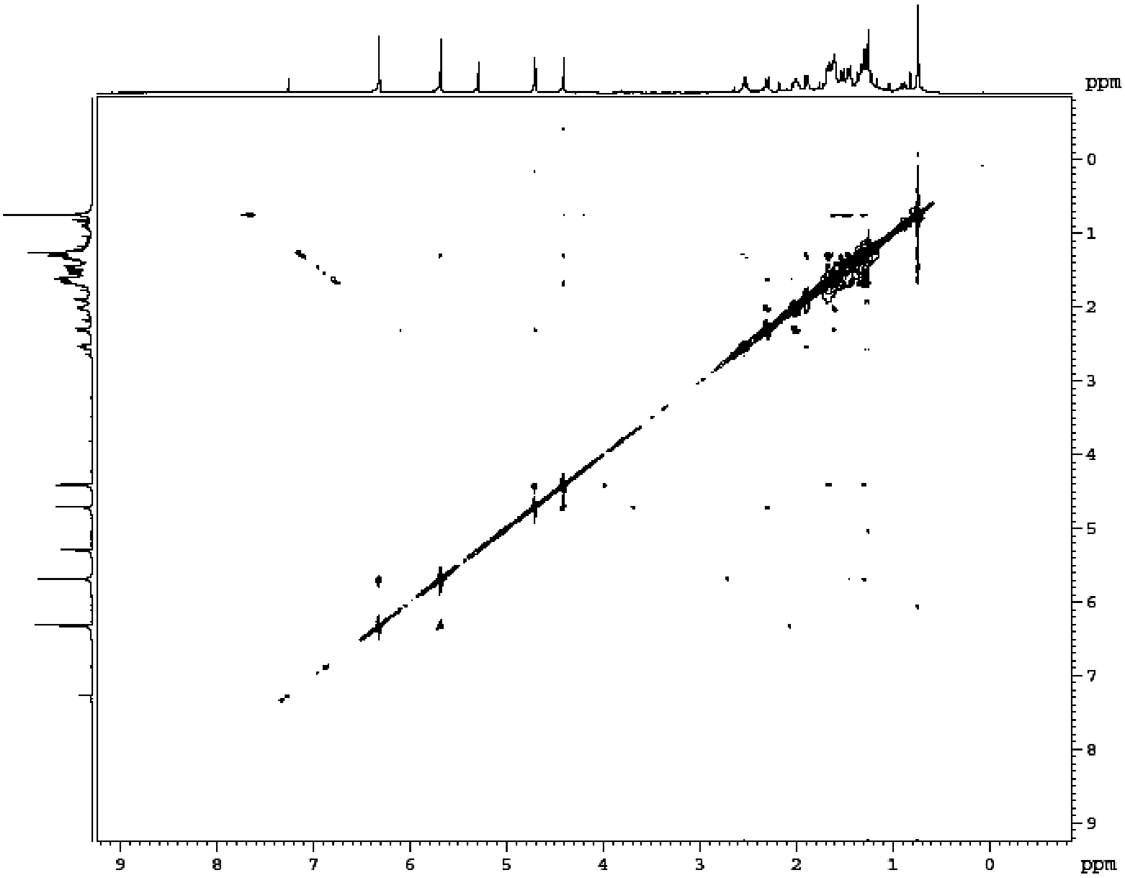

NOESY spectrum of costic acid (0.7–3.1 ppm)

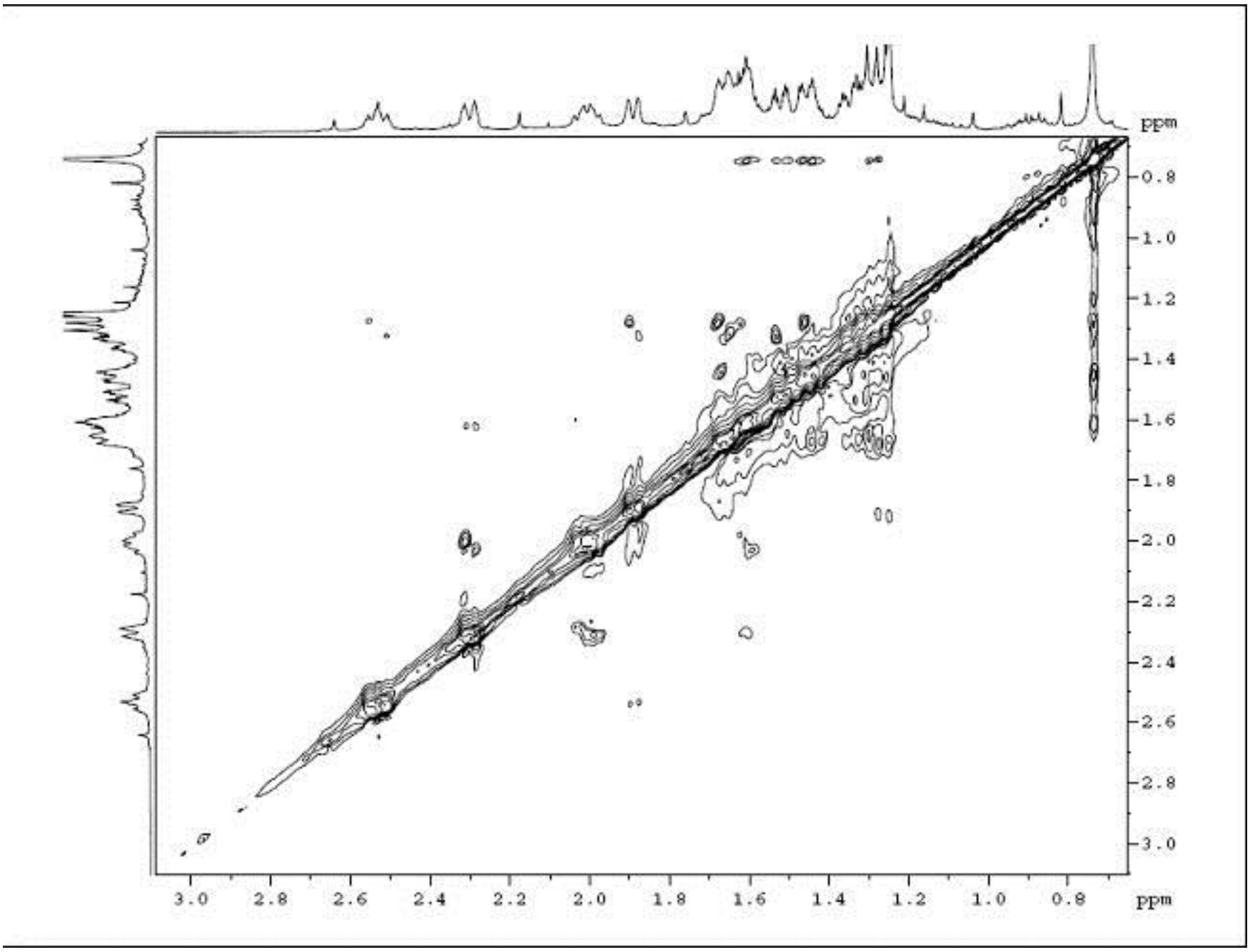

FTIR spectrum of costic acid

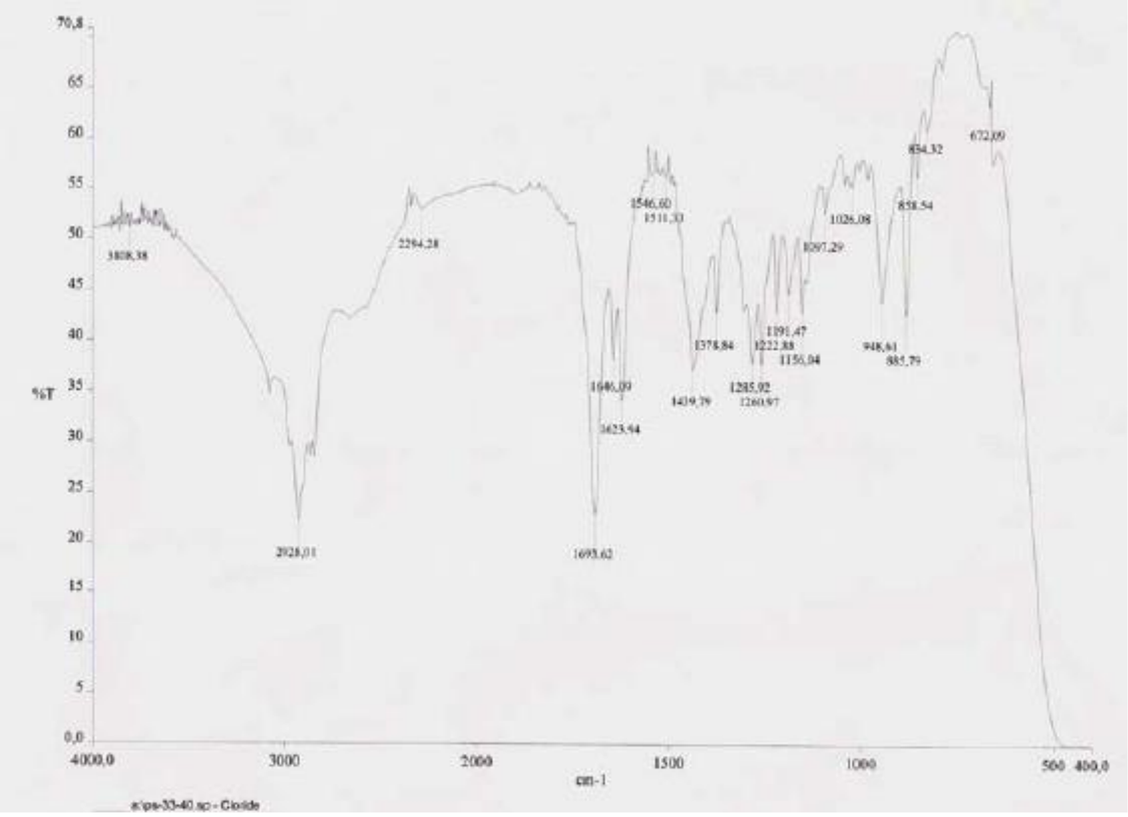

Mass spectrum of costic acid

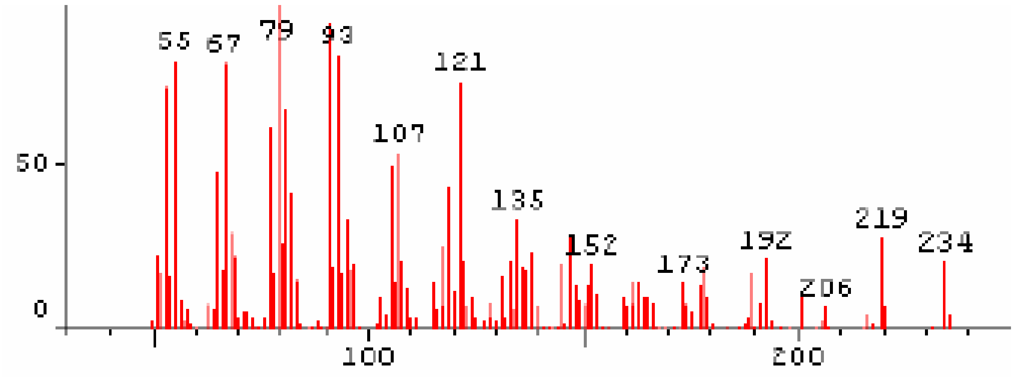

Supplement: File 1 — NMR, IR and MS spectra of costic acid. [file Beilstein_J_Org_Chem-13-952-s001.pdf]
